# Room Temperature Fabrication of Stable, Strongly Luminescent Dion–Jacobson Tin Bromide Perovskite Microcrystals Achieved through Use of Primary Alcohols

**DOI:** 10.3390/nano11102738

**Published:** 2021-10-16

**Authors:** Jinsong Qi, Shixun Wang, Arsenii Portniagin, Stephen V. Kershaw, Andrey L. Rogach

**Affiliations:** Department of Materials Science and Engineering, City University of Hong Kong, 83 Tat Chee Avenue, Hong Kong SAR 999077, China; jinsongqi2-c@my.cityu.edu.hk (J.Q.); shixuwang3-c@my.cityu.edu.hk (S.W.); aportniag2-c@my.cityu.edu.hk (A.P.); skershaw@cityu.edu.hk (S.V.K.)

**Keywords:** Dion–Jacobson perovskites, tin bromide perovskites, photoluminescence, crystallization, primary alcohols

## Abstract

Lead-free two-dimensional metal halide perovskites have recently emerged as promising light-emitting materials due to their improved stability and attractive optical properties. Herein, a facile room temperature wet milling method has been developed to make Dion–Jacobson (DJ) phase ODASnBr_4_ perovskite microcrystals, whose crystallization was accomplished via the aid of introduced primary alcohols: ethanol, butanol, pentanol, and hexanol. Due to the strong intermolecular hydrogen bonding, the use of ethanol promoted the formation of non-doped ODASnBr_4_ microcrystals, with an emission peaked at 599 nm and a high photoluminescence quantum yield (PL QY) of 81%. By introducing other primary alcohols with weaker intermolecular hydrogen bonding such as butanol, pentanol, and hexanol, [SnBr_6_]^4−^ octahedral slabs of the DJ perovskite microcrystals experienced various degrees of expansion while forming O–H…Br hydrogen bonds. This resulted in the emission spectra of these alcohol-doped microcrystals to be adjusted in the range from 572 to 601 nm, while keeping the PL QY high, at around 89%. Our synthetic strategy provides a viable pathway towards strongly emitting lead-free DJ perovskite microcrystals with an improved stability.

## 1. Introduction

Metal halide perovskites have recently emerged as a popular material owing to their attractive optical properties, such as tunable absorption and photoluminescence (PL), and high defect tolerance [1]. The so-called three-dimensional (3D) perovskites are represented by a general formula ABX_3_, where A is a monovalent cation such as CH_3_NH_3_^+^ (methylammonium), HC(NH_2_)_2_^+^ (formamidinium) or Cs^+^; B is a divalent metallic cation (typically Pb^2+^, but could be also Ge^2+^, Sn^2+^, etc.), and X is a monovalent halide anion (Cl^−^, Br^−^, I^−^, or their combination in mixed halide alloys). There have been already promising reports of their application for various kinds of devices, such as solar cells [2], light-emitting diodes [3], and photodetectors [4]. However, the toxicity of lead which still remains the main constituent of the large variety of the reported metal halide perovskites, and their poor stabilities towards polar solvents and light irradiation greatly limit the practical application of 3D perovskites.

Even more recently, two-dimensional (2D) perovskite structures, namely Ruddlesden–Popper (RP) and Dion–Jacobson (DJ) perovskites, have ignited the interest of scientists owing to their higher structural stability and at the same time their rather promising PL performance [5,6,7]. These 2D structures retain the main structural feature of a 3D perovskite lattice, in which octahedral metal halide units are connected by shared corners. At the same time, the interlayers between the planes of interconnected octahedra of the 2D perovskites are populated with monovalent or divalent long-chain organic cations, which form either the RP or DJ phases, respectively. Unlike the RP perovskites, DJ perovskites do not experience van der Waals interactions within their interlayers, because their extended divalent organic cations are connected with inorganic perovskite layers by forming hydrogen bonds at both ends, which often renders them with comparatively higher structural stability [8,9,10,11]. These DJ perovskites have already found application in solar cells [10] and light-emitting diodes [12], but their external quantum efficiency still has a lot of room for improvements. Thus, synthetic efforts towards strongly luminescent DJ perovskites constitute an important research task. Our group has recently introduced small molecules acting as acidic proton donors, such as dichloromethane and chloroform, which served as molecular dopants to improve the crystallinity of DJ phase tin bromide ODASnBr_4_ perovskite microcrystals (ODA stands for 1,8-octanediamine), achieving remarkable PL quantum yields (QY) approaching 90% [13]. Moreover, primary alcohols were also found to be able to form hydrogen bonds with DJ phase ODASnBr_4_ microcrystals, and in addition helped to remove byproducts formed during the conventional saturation recrystallization process [14]. However, while applied as a post-synthetic treatment agent, ethanol (EtOH) required very long treatment times or higher temperatures to break intermolecular O–H…O hydrogen bonds and form O–H…Br hydrogen bonds with the perovskite lattice [14].

Herein, by using a mixture of perovskite precursors and EtOH, DJ phase non-doped ODASnBr_4_ perovskite microcrystals denoted as ODASnBr_4_(EtOH) were prepared at room temperature and showed a high PL QY of 81% for emission peaked at 599 nm. By using longer-chain primary alcohols, 1-butanol (BuOH), 1-pentanol (PeOH), or 1-hexanol (HeOH), which were able to form O–H…Br hydrogen bonds with the perovskite lattice, doped ODASnBr_4_[alcohol] microcrystals with PL QYs reaching 89% and PL peaks adjustable between 572 nm and 601 nm have been observed. This study is a logical continuation of our work to develop the facile synthesis of strongly emitting lead-free DJ low-dimensional perovskites.

## 2. Materials and Methods

### 2.1. Materials

Tin (II) bromide (SnBr_2_, 99.9%) and 1,8-octanediamine (ODA, 98%) were purchased from Sigma Aldrich, Burlington, MA, USA. Hydrogen bromide (HBr, 48% solution) was purchased from Aladdin, Shanghai, China. Ethanol (EtOH, 99%), 1-propanol (PrOH, 99%), 1-butanol (BuOH, 99%), 1-pentanol (PeOH, 99%), and 1-hexanol (HeOH, 99%) were purchased from Duksan Pure Chemicals Co., Ltd, Ansan-si, Korea.

### 2.2. Synthesis of ODASnBr_4_(EtOH) and ODASnBr_4_[Alcohol] Perovskite Microcrystals

The 0.4 mmol SnBr_2_, 0.4 mmol ODA, 0.2 mL HBr, and 0.5 mL BuOH, PeOH, or HeOH were successively added into a mortar and ground together to prepare alcohol-doped ODASnBr_4_[BuOH], ODASnBr_4_[PeOH] and ODASnBr_4_[HeOH] perovskite microcrystals, respectively. Dopant-free ODASnBr_4_, denoted as ODASnBr_4_(EtOH) could also be made in a similar way using EtOH, but they would degrade within 10 min due to oxidation of Sn(II) in the air, because EtOH does not function as a molecular dopant under such a synthesis condition [14]. Therefore, in order to prepare ODASnBr_4_(EtOH) microcrystals, respective precursors were mixed together in a 2 mL glass vial, which was sealed and shook for 5 min. All samples were rinsed with EtOH at room temperature in order to remove any non-reacted precursors and undesired impurities [14], and dried in the vacuum box for further use.

### 2.3. Characterization

Powder X-Ray diffraction (XRD) patterns were collected on a Rigaku SmartLab X-ray diffractometer, Tokyo, Japan. Optical diffuse-reflectance spectra were collected on a Shimadzu UV 3600 UV/visible/IR spectrophotometer, Kyoto, Japan with an integrating sphere accessory. PL and PL excitation (PLE) spectra, as well as time-resolved PL decays were measured on an FLS920P spectrometer (Edinburgh Instruments, Livingston, UK). Absolute PL QYs were measured with the aid of an integrating sphere with its inner face coated with BENFLEC™ (Edinburgh Instruments, Livingston, UK). X-ray photoelectron spectroscopy (XPS) measurements were performed on a PHI model 5802 instrument (ULVAC-PHI, Inc., Kanagawa, Japan). Fourier-transform infrared (FTIR) spectra were collected on a Perkin Elmer FTIR spectrophotometer (Perkin Elmer, Waltham, MA, USA). Raman spectra were collected on a WITec Alpha300 R confocal Raman imaging system (WITec Wissenschaftliche Instrumente und Technologie GmbH, Ulm, Germany) equipped with a 532 nm laser. An FEI Quanta 250 e-scanning electron microscope (SEM) (Thermo Fisher Scientific Inc., Waltham, MA, USA) was used to study the morphology and elemental composition of the samples.

## 3. Results and Discussion

**Figure 1:**
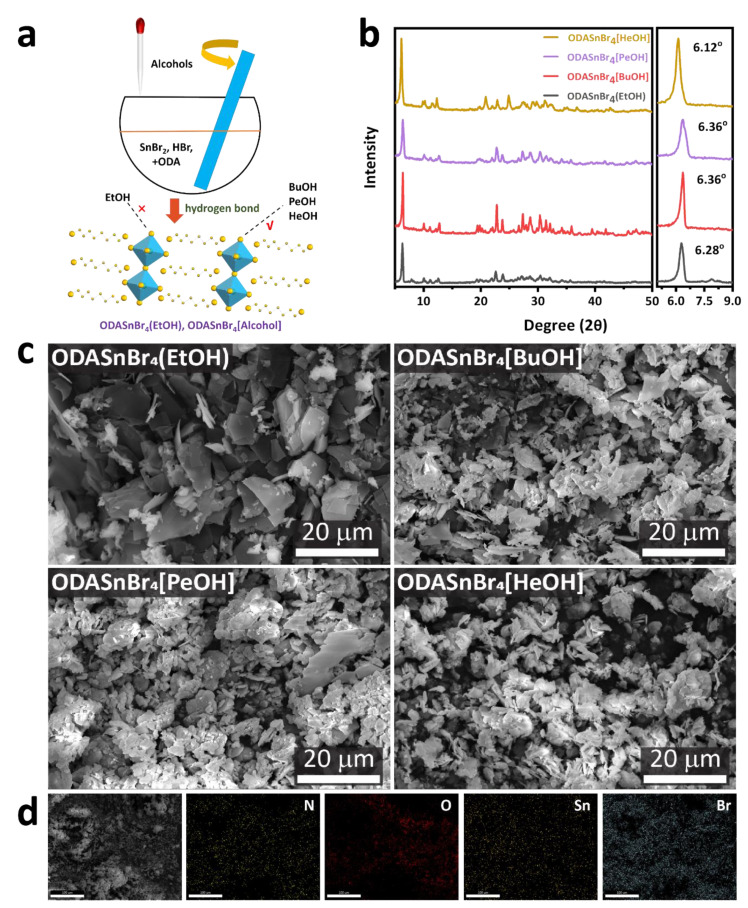
(**a**) Schematics of the ODASnBr_4_(EtOH) and ODASnBr_4_[alcohol] fabrication processes. (**b**) Powder XRD spectra and (**c**) SEM images of ODASnBr_4_(EtOH), ODASnBr_4_[BuOH], ODASnBr_4_[PeOH], and ODASnBr_4_[HeOH] perovskite microcrystals, (**d**) SEM image (left) and EDS elemental mapping images of ODASnBr_4_[HeOH] microcrystals for constituent elements N, O, Sn and Br (scale bar: 20 µm).

After mixing ODA, SnBr_2_, and HBr precursors with different primary alcohols in a mortar, a room temperature (25 °C) wet grinding process was conducted to prepare ODASnBr_4_ perovskite microcrystals as shown in Figure 1a. Powder XRD patterns were collected to compare the structural characteristics of perovskites produced using different primary alcohols. As shown in Figure 1b, ODASnBr_4_(EtOH), ODASnBr_4_[BuOH], ODASnBr_4_[PeOH], and ODASnBr_4_[HeOH] microcrystals exhibited a dominant peak located at 6.28°, 6.36°, 6.36°, and 6.12°, respectively, which corresponds to the diffraction from (002) perovskite lattice planes. The (002) lattice plane of ODASnBr_4_[HeOH] underwent a shift of 0.24^o^ towards lower 2θ as compared with that of the BuOH and PeOH doped ODASnBr_4_ microcrystals, revealing a higher level of lattice expansion between octahedron slabs caused by the longer carbon chain of HeOH. Notably, as compared with ODASnBr_4_[BuOH] and ODASnBr_4_[PeOH], the diffraction peak of ODASnBr_4_(EtOH) at 6.28° still experienced a shift of 0.08° towards lower 2θ, even though EtOH possesses a shorter carbon chain than BuOH and PeOH. This slight lattice dilation was due to the presence of byproducts (ODA·2HBr), which were physically inserted in the perovskite lattices [15,16] which also showed up through the presence of a broad diffraction peak at 8.4^o^ (Figure 1b). Importantly, XRD patterns have also shown that the room temperature wet grinding method applied here could efficiently suppress the crystallization and growth of any byproducts while leading exclusively to the formation of thermodynamically favored DJ phase perovskites. As such, the once dominating diffraction peak of impurities at around 8.4° (reported in our previous related study [14]) was drastically reduced in ODASnBr_4_(EtOH) and absent completely in all the other ODASnBr_4_[alcohol] microcrystals.

SEM images of the obtained perovskite microcrystals are shown in Figure 1c. ODASnBr_4_(EtOH) microcrystals possess a typical 2D sheet-like morphology with a lateral length of around 10 µm. The size of the sheets decreased while using long-chain alcohols BuOH, PeOH, and HeOH, probably due to the longer chain alcohols that may favor stronger hydrogen bonding through the electromeric effect of the alkyl portion of the molecule pushing more electron density back towards the OH group, unlike for smaller EtOH molecules dominated by intermolecular O–H…O hydrogen bonds [16]. Figure 1d provides energy-dispersive X-ray spectroscopy (EDS) elemental mapping images for the representative case of ODASnBr_4_[HeOH] microcrystals, showing the presence of N, O, Sn, and Br elements, with atomic ratios of 52%, 21%, 5%, and 22%, respectively. Excess oxygen and nitrogen may originate from the presence of alcohol dopants and eventually some residual amine precursors.

**Figure 2:**
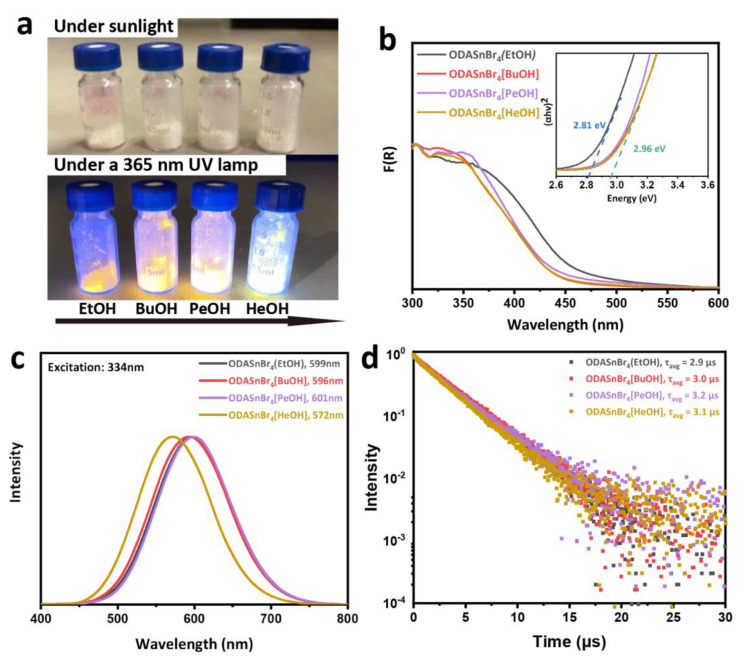
(**a**) Photograph of the ODASnBr_4_(EtOH), ODASnBr_4_[BuOH], ODASnBr_4_[PeOH], and ODASnBr_4_[HeOH] perovskite microcrystals taken under sunlight and under a 365 nm UV lamp; (**b**) absorption spectra represented by Kubelka–Munk function, F(R); the inset shows the corresponding Tauc plots used to determine the optical bandgaps, (**c**) normalized PL spectra, and (**d**) normalized PL decay curves of these four samples.

Figure 2a shows photographs of four kinds of perovskite microcrystals taken under sunlight and a 365 nm UV lamp, respectively. Powdered samples appear white under sunlight, and emit strong orange light under UV excitation. Absolute PL QYs of ODASnBr_4_(EtOH), ODASnBr_4_[BuOH], ODASnBr_4_[PeOH], and ODASnBr_4_[HeOH] perovskite microcrystals were determined to be 81%, 89%, 88%, and 89%, outperforming most other low-dimensional perovskites (Table 1). From the absorption spectra and the respective Tauc plots derived from these, optical bandgaps of the samples have been determined (Figure 2b). Three samples ODASnBr_4_[BuOH], ODASnBr_4_[PeOH], and ODASnBr_4_[HeOH] microcrystals showed similar bandgaps of 2.96 eV, which were blue-shifted from the value of 2.81 eV for ODASnBr_4_(EtOH) due to the slight lattice expansion caused by these alcohol dopants and the ODA·2HBr impurities [14]. We notice that the XRD patterns in Figure 1b and absorption spectra in Figure 2b do not match fully with those of the post-treated ODASnBr_4_[alcohol] microcrystals reported in our previous study [14], which may be due to the different amount of crystalized/uncrystallized impurities that are mixed in with the perovskite lattices, considering the broad XRD peak at around 30°.

As shown in Figure 2c, ODASnBr_4_(EtOH), ODASnBr_4_[BuOH], and ODASnBr_4_[PeOH] microcrystals excited at 334 nm exhibited PL peaks at 599, 596, 601 nm, respectively, while the PL maximum of ODASnBr_4_[HeOH] underwent a much larger blue shift to 572 nm. Such a strong blue shift in the latter case may be determined by a greatly increased distance between perovskite slabs in the ODASnBr_4_[HeOH] lattice, according to their XRD patterns, which influenced the radiative recombination channels. Time-resolved PL decays of the four samples are shown in Figure 2d. Average PL lifetimes (*τ*_avg_) were calculated from these decays by using the following equation [17]: $\tau_{\mathrm{avg}}=\frac{\;\int_{0}^{\infty }tI\left(t\right)dt}{\int_{0}^{\infty }I\left(t\right)dt}$ and were found to be 2.9, 3.0, 3.2, and 3.1 μs for the ODASnBr_4_(EtOH), ODASnBr_4_[BuOH], ODASnBr_4_[PeOH], and ODASnBr_4_[HeOH], respectively. Such long PL decay times, alongside with broad PL profiles with large Stokes shifts are characteristic for self-trapped exciton states (STE) of the 2D tin halide perovskites that possess a soft lattice and strong electron–phonon interaction [18].

**Table 1:** Comparison of PL characteristics of various low-dimensional metal halide perovskites.

Materials	PL Peak [nm]	PLQY [%]	Year	Ref.
Cs_4_PbBr_6_	515	54	2017	[19]
(C_4_N_2_H_14_Br)_4_SnBr_3_I_3_	582	85	2017	[20]
(C_9_NH_20_)_2_SnBr_4_	695	46	2018	[21]
[(PEA)_4_SnBr_6_](PEA)	566	89.5	2020	[22]
(ODA)Sn_2_I_6_	631	36	2020	[7]
PDAm-Rb	485	68.8	2021	[23]
ODASnBr_4_[CFM/DCM]	570–598	88 ± 4	2021	[13]
ODASnBr_4_[alcohol]	611–616	85 ± 2	2021	[14]
ODASnBr_4_(EtOH) ODASnBr_4_[alcohol]	599	81	2021	This work
	572–601	88 ± 1	2021

**Figure 3:**
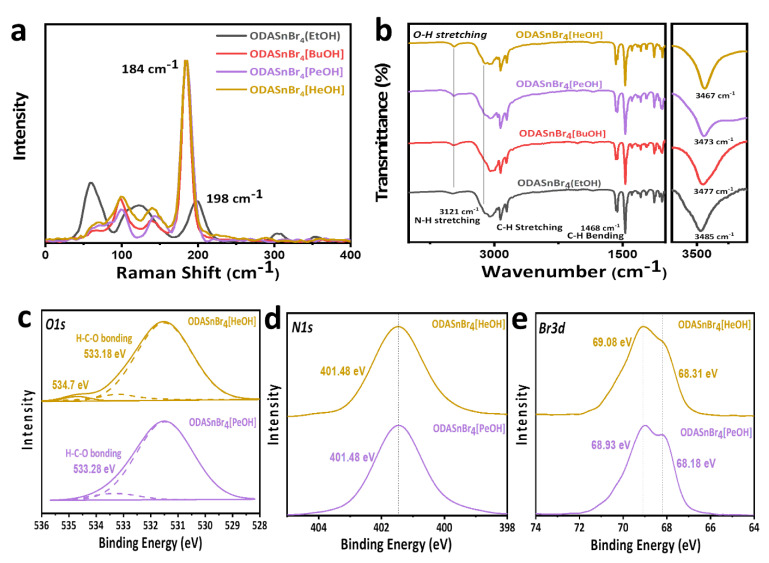
(**a**) Raman spectra and (**b**) FTIR spectra of ODASnBr_4_[EtOH], ODASnBr_4_[BuOH], ODASnBr_4_[PeOH], ODASnBr_4_[HeOH]. (**c**–**e**) XPS spectra of (**c**) O1s, (**d**) N1s, (**e**) Br3d core-levels of ODASnBr_4_[PeOH], and ODASnBr_4_[HeOH] perovskite microcrystals.

Raman spectra (Figure 3a) of the four samples were compared with the purpose of evaluating the lattice expansion degree related to the alcohol dopants and the [SnBr_6_]^4−^ octahedra of the perovskite lattice. ODASnBr_4_[BuOH], ODASnBr_4_[PeOH], and ODASnBr_4_[HeOH] microcrystals all showed a dominant signal at 184 cm^−1^ corresponding to the Sn–Br symmetric vibration, indicating a similar degree of lattice expansion for the [SnBr_6_]^4−^ octahedra caused by these alcohol dopants. This observation stresses that the changes of the (002) lattice planes for the ODASnBr_4_[alcohol] samples primarily originate from the dilated A-site of the perovskite lattices due to the presence of alcohol dopants or long-chain impurities. The same peak for ODASnBr_4_(EtOH) microcrystals was located at 198 cm^−1^ due to the absence of O–H…Br hydrogen bonds between EtOH and [SnBr_6_]^4−^ octahedral. Furthermore, the Raman signal at around 64 cm^−1^, corresponding to protonated amine impurities (ODA·2HBr) [14], was greatly suppressed in ODASnBr_4_[alcohol] samples, except for ODASnBr_4_(EtOH). It is noted that the broad Raman signal at around 120 cm^−1^ could arise from a combination of Br−Sn−Br asymmetric bending (~100 cm^−1^) and the Sn−Br asymmetric stretch (142 cm^−1^). Therefore, the different degrees of lattice expansion on the XRD patterns could primarily be attributed to the different lengths of alcohols and any long-chain byproducts (ODA·2HBr) for ODASnBr_4_[alcohol] and ODASnBr_4_(EtOH) samples, respectively.

Fourier transform infrared (FTIR) spectra provided in Figure 3b show that the frequency corresponding to the O–H stretching vibration gradually shifted from 3485 cm^−1^ for ODASnBr_4_(EtOH) to 3477 cm^−1^ for ODASnBr_4_[BuOH], 3473 cm^−1^ for ODASnBr_4_[PeOH], and 3467 cm^−1^ for ODASnBr_4_[HeOH]. This shift can be attributed to the increasing O–H bond length due to stronger O–H…Br hydrogen bonding between longer chain alcohol dopants and [SnBr_6_]^4−^ octahedra influenced by the electromeric effect of the alkyl portion of the molecule. However, if we assume a similar degree of distortion of [SnBr_6_]^4−^ octahedra in the [alcohol]-doped samples, this cannot explain the large difference in the positions of the PL maxima for ODASnBr_4_[BuOH] and ODASnBr_4_[PeOH] on one hand, and ODASnBr_4_[HeOH] on the other hand (Figure 2c). Thus, X-ray photoelectron spectroscopy (XPS) has been performed on two samples ODASnBr_4_[PeOH] and ODASnBr_4_[HeOH] in order to reveal more details of their coordination. As can be seen from the XPS O1s core-level spectra provided in Figure 3c, ODASnBr_4_[HeOH] microcrystals have a slightly lower C–O binding energy (533.18 eV) as compared with ODASnBr_4_[PeOH] (533.28eV), which may induce stronger O–H…Br hydrogen bonding between HeOH and [SnBr_6_]^4−^ octahedra; the peak at 534.7 eV which appears in ODASnBr_4_[HeOH] may be related to oxygen from water molecules [24,25]. According to the N1s spectra shown in Figure 3d, ODASnBr_4_[PeOH] and ODASnBr_4_[HeOH] have similar binding energy for the –NH_3_^+^ group at 401.48 eV, which rules out the coordination between the protonated amine group and the δ^−^ polarized oxygen atom from the alcohol dopants. From Figure 3e, the binding energy of the Br3d_3/2_ core levels for ODASnBr_4_[HeOH] increased from 68.93 to 69.08 eV, and for Br3d_5/2_ core levels from 68.18 to 68.31 eV, as compared to ODASnBr_4_[PeOH], which is consistent with the lower C–O binding energy of the former.

**Figure 4:**
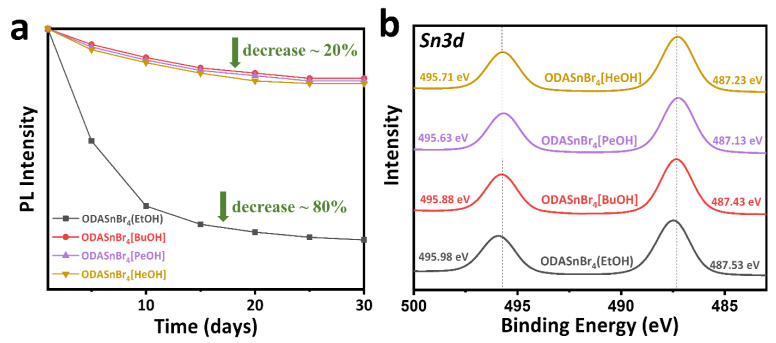
(**a**) Change of PL intensity and (**b**) XPS spectra of the Sn3d core level of ODASnBr_4_(EtOH), ODASnBr_4_[BuOH], ODASnBr_4_[PeOH], and ODASnBr_4_[HeOH] perovskite microcrystals followed for a period of up to 30 days, for the samples stored under ambient conditions in sealed glass vials with humidity above 50% when bottled.

All of the [alcohol]-doped tin bromide perovskite microcrystals produced in this work showed remarkably high stabilities of their PL intensities, when stored under ambient conditions in sealed glass vials with humidity above 50% at the point of bottling. As shown in Figure 4a, ODASnBr_4_[BuOH] and ODASnBr_4_[PeOH] and ODASnBr_4_[HeOH] maintained around 80% of their initial intensity for up to 30 days. ODASnBr_4_(EtOH) microcrystals, in contrast, were more susceptible to oxidation, as their PL intensity dropped to only 20% of the initial value during the storage for 30. This can be contrasted with the reasonably high thermal stability of the, to date, more comprehensively explored ODASnBr_4_ microcrystals probably due to the absence of molecular dopants [14]. XPS Sn3d core-level spectra measured on perovskite microcrystals after 30 days storage (Figure 4b) showed that the binding energy corresponding to Sn3d_5/2_ changed from 487.53 eV for ODASnBr_4_(EtOH) to 487.43 eV for ODASnBr_4_[BuOH], 487.13 eV for ODASnBr_4_[PeOH], and 487.23 eV for ODASnBr_4_[HeOH], indicating that perovskite microcrystals were better protected from oxidation after incorporating long-chain primary alcohol dopants.

## 4. Conclusions

Strongly luminescent lead-free DJ phase ODASnBr_4_ perovskite microcrystals were produced in this work using a room temperature, wet milling method with the aid of primary alcohols: ethanol, 1-butanol, 1-pentanol, and 1-hexanol. Dopant-free ODASnBr_4_(EtOH) perovskite microcrystals were formed because EtOH could not form O–H…Br hydrogen bonds with the perovskite lattices under this synthetic condition while providing a homogeneous reaction environment like octadecene in classic colloidal synthesis. When using BuOH, PeOH, and HeOH, doped ODASnBr_4_[alcohol] microcrystals were obtained due to the formation of O–H…Br hydrogen bonding between [SnBr_6_]^4−^ octahedra. Perovskite microcrystals synthesized by this method showed a strong PL emission (PL QY over 80%) tunable in the range of 572 nm to 601 nm. The PL intensity of ODASnBr_4_[alcohol] microcrystals could be maintained at around 80% of their initial PL intensity after being stored in a sealed glass bottle for a month, due to their improved crystallinity and remarkable stability against oxidation provided by the primary alcohol dopants.

## Data Availability

Data can be available upon request from the authors.
